# Demographic and socio-economic factors associated with multiple health risk behaviours among adolescents in Serbia: a cross sectional study

**DOI:** 10.1186/s12889-015-1509-8

**Published:** 2015-02-19

**Authors:** Katarina Boričić, Snežana Simić, Jelena Marinković Erić

**Affiliations:** Center for Health Promotion, Institute of Public Health of Serbia “Dr Milan Jovanovic Batut”, 5 Dr Subotic Street, 11000 Belgrade, Republic of Serbia; Institute of Social Medicine, Faculty of Medicine, University of Belgrade, Belgrade, Republic of Serbia; Institute of Medical Statistics and Informatics, Faculty of Medicine, University of Belgrade, Belgrade, Republic of Serbia

**Keywords:** Risk-taking, Socioeconomic factors, Demographic factors, Adolescent

## Abstract

**Background:**

The aim of this study was to examine the relationships between demographic and socioeconomic characteristics and engaging in multiple risk behaviours among adolescents in Republic of Serbia.

**Methods:**

This study presents a cross sectional study of 683 adolescents aged 15 to 19 attending high school. The database from the 2006 National Health Survey was used. As a measure of demographic and socio-economic characteristics: age, type of settlement, family structure, having one’s own room, school success and the household wealth index were used. Multivariate logistic regression model was performed.

**Results:**

Boys were more than twice as likely to engage in multiple risk behaviours than girls. Adolescents who were older (OR = 5.82, 95% CI = 3.21–10.54, boys; OR = 3.76, 95% CI =1.77–7.99, girls) and adolescents who achieved low or moderate (OR = 1.82, 95% CI = 1.02–3.26, boys; OR = 3.36, 95% CI =1.51–7.44, girls) school success had significantly higher risk than younger ones and those with high school success. Also, boys who came from a richer class households (OR = 3.14, 95% CI =1.02–9.66) and girls from incomplete family (OR = 5.07, 95% CI = 2.06–12.50) had higher risk than boys from the poorest households and girls from complete family.

**Conclusions:**

Further preventive interventions in Serbia should be gender and age specific, oriented towards older adolescents, those who have low or moderate school success, boys from richer class households and girls who live in incomplete families.

## Background

Adolescents’ health presents a multi-value for themselves, their families and communities and a basis for sustainable development of every society and it depends on the efforts being made to preserve and improve it [1].

The category of adolescents covers the ages 10–19. Adolescents may, according to health indicators, be considered healthier than all other age groups, so the analysis of their health status should be health-oriented. However, due to many specific features of this period (sexual and psycho-physical development) and risk to take on dangerous behaviours that may jeopardize health it is necessary to assess the presence of these risk behaviours and to undertake adequate health educational interventions to promote healthy behaviour and life styles [2].

Risky behaviour can be defined as “voluntary behaviour that follows the existence of a specific objective and/or subjective degree of risk” or the “specific form of behaviour which has been shown to increase susceptibility to specific diseases or health disorders”. In 1990, Irwin stated that these forms of behaviour should be called “risk-taking behaviour,” because it involves adolescents who knowingly and willingly engage in situations where the risk is certain, and the outcome is unknown, but with a high probability to be negative for health [3].

Generally, health risk behaviours tend to cluster together [4-6], and, in some adolescents, the clustering is sufficiently strong to develop a “risk behaviour syndrome” [7]. Risk behaviours in adolescents are multicausal behaviours. In this sense, it is considered that the effects of individual biological, psychological and sociological conditions, especially the family situation, and peer influence are important [8,9].

The state of health of adolescents in the Republic of Serbia does not differ significantly in comparison to that of adolescents in the world, but there are, nonetheless, certain specificities. Namely, the last decade of 20th century in Serbia was marked by wars, economic sanctions of the international community and negative consequence which they caused and this in turn led to a disintegration of all segments of the society, including the family and school environment, as well as health care. Adolescents grew up in isolation, without appropriate social care, swamped by images of violence through the media, finding themselves in poverty they had not caused [2].

Most the previous studies in Serbia were exclusively focused on single risk factors [10-13]. This is the first study investigating the prevalence of concurrent health risk behaviours and its association with demographic and socio-economic determinants of health among adolescents in Serbia. Considering that, the results of this study can make a significant contribution to the provision of information to professional public and decision makers, especially on the importance of socio-economic and demographic determinants in the creation of public health programs whose implementation would contribute to reducing inequalities in adolescents’ health.

The aim of this study was to examine the relationships between demographic characteristics and socio-economic status and engaging in multiple risk behaviours among adolescents in the Republic of Serbia.

## Methods

### Population and sampling

This study presents a cross sectional study of a sample of 683 adolescents aged 15 to 19 attending high school. The study used a database from the 2006 Health Survey of the Republic of Serbia (without data for Kosovo and Metohija), which was carried out by the Ministry of Health of the Republic of Serbia with financial and professional support of the World Bank, the World Health Organization Regional Office for Europe (country office Serbia) and the Institute of Public Health of Serbia ‘Dr Milan Jovanovic Batut’ [14].

The 2006 Health Survey of the Republic of Serbia provided statistically reliable estimates of the health indicators at the national level and at the levels of six geographic regions: Vojvodina, Belgrade, West, Central, East and South-East Serbia. By their further division into urban and rural areas, twelve areas were identified as the main sampling strata. The sample was selected in two stages. The primary stage units were 675 enumeration areas from the Census of 2002 in Serbia, selected on the basis of probability proportional sampling. Second stage units were households, selected by simple random sampling without replacement. After updating within each selected census enumeration areas, 10 households and 3 replacement households from the household list were chosen. The replacement households were interviewed only if some of the first 10 households were not found. In the case that a household refused to be interviewed, a replacement household was not contacted In this way, 7673 selected households were made sampling frame and observation units were all members of the selected households.

Out of 7673 households randomly selected for the sample, the members of 6156 households were interviewed. The household response rate was 86.5%. In selected households, 683 adolescents aged 15 to 19 attending high school were identified.

Cross-sectional data were weighted to represent the Serbian population in 2002. The weights were adjusted by population projections for 2006 based on the vital statistics (birth and death rate).

### Ethical issues

Informed consent was obtained from all respondents. The study was approved by the Review Board of the Ministry of Health of Serbia and the Institute of Public Health of Serbia.

### Instruments

Three types of questionnaires were used to collect data: household questionnaire, questionnaire for children and adolescents aged 7–19 years (face to face) and self-administered questionnaire for children and adolescents aged 12–19 years. Five questions that were related to demographic characteristics and socio-economic status of adolescents of the 81 questions from a face-to-face questionnaire and 6 questions that related to various forms of risky behaviour of the 66 questions from a self-questionnaire for children and adolescents aged 12 to 19 were used. Socio-economic status was measured by calculating the demographic and health survey wealth index (wealth index) on the basis of answers to 9 questions from the household questionnaire that included 30 questions.

Data collection process was standardized in order to ensure the quality of data collection and that a consistent methodology would be used. Before the start of interviewing, training for 201 interviewers was conducted in the form of two-day workshops. The obligation of the interviewers was to interview all household members.

### Data

As a measure of demographic and socio-economic characteristics: age (categorized into two age groups: one – 15–16 and two – 17–19), type of settlement (one – non-urban and two – urban), family structure (one –complete: with both biological parents, with one biological parent and stepmother/stepfather or with caregivers, two – incomplete: with one biological parent, be alone or with grandparents), having one’s own room (one – no and two – yes), school success (categorized into three groups: one – high (excellent, very good), two – moderate or low (good, sufficient, insufficient), and the household wealth index (one – poorest, two – poorer, three – middle, four – richer and five – richest class) were used. Assets included in computing household wealth index were number of bedrooms per household member, material used for floor, roof and walls of the house type of drinking water source and sanitation facilities, source of energy used for heating, possession of colour TV, mobile phone, refrigerator, personal computer, washing machine, dishwasher, air conditioning, central heating, car and internet access. The distribution of the household population by household wealth index was performed on 5 categories by 20% quintiles [15].

In secondary schools in Serbia, the evaluation of success in school for every student is performed using a five-point grading scale and it is assessed by averaging his grades in all subjects. At the end of the school year, final grades for each subject are calculated from those given at the end of each semester and they are determined by the following ranges: 5 (excellent) is given for an average of 4.50 to 5.00; 4 (very good) is given for an average of 3.50 to 4.49; 3 (good) is given for an average of 2.50 to 3.49; 2 (sufficient) the lowest passing grade is given for an average of 2.00 to 2.49; 1 (insufficient) the lowest possible grade, and the failing one, is given if the student does not have grade of at least 2 in each topic of the course.

Data on the prevalence of the single health risk among adolescents were assessed by the responses of adolescents about smoking at least one cigarette per day during the previous month, drinking any alcoholic beverage from the list of drinks: beer, wine, spirits, liqueur cocktail at least one day during the previous month, taking non-prescription tablets (anxiolytics, analgesics, amphetamine etc.) during the previous month, having the experience of casual sexual intercourse during the last 12 months, having first sexual intercourse before the age of 16 and bullying others. In order to determinate risk behaviour that related to bullying somebody, young people were asked whether they had taken part in insults, humiliation or physical harassment of another person during their lifetime. For further analysis, the adolescents were divided into three categories: no risk, one risk, and two or more health risk behaviours.

### Statistics

Data were analysed by descriptive and inferential statistics. At the level of inferential statistics, nonparametric chi-square test was used for testing the statistical significance of the difference between the variables and multivariate logistic regression was used for statistical modeling separately for boys and for girls.

Distribution of boys and girls and their differences according to demographic and socio-economic status variables, single or various models of multiple health risk behaviours were examined by chi-square test or Fisher’s exact test. Also, distribution of boys and girls with various models of health risk behaviours (no risk, one risk, and two or more health risk behaviours) and their differences according to demographic and socio-economic status variables were examined by chi-square test. Finally, multivariate logistic regression model was used to determine predictors of concurrent health risk behaviours. The dependent variable was engaging in multiple risk behaviours (two or more risk behaviours vs no risk behaviours). All analyses were done separately for boys and girls. The odds ratios (ORs) with their corresponding 95% confidence intervals (CIs) were adjusted for age, type of settlement, household wealth index, family structure, having one’s own room and school success.

Statistical package statistical software package SPSS 17 was used for data analysis. Differences were considered statistically significant at P < 0.05.

## Results

### Sociodemographic factors and household wealth index

There were more girls (51.6%) than boys (48.4%) in the sample. The mean age of the boys was 16.29 ± 1.15 and that of the girls was 16.32 ± 1.12. About sixty percent of them came from an urban environment during the time of the survey although the boys were slightly more likely to live in urban environment than the girls. More than ten percent (12.2%) adolescents lived in the poorest households, while the majority of them (26.5%) came from a household with the household wealth index four (richer adolescents).

Nearly three quarters of adolescents (72.3%) had their own room, while more than four fifths of them (88.4%) lived in complete families, slightly more boys than girls. Twice as many adolescents (63.8%) achieved high success in school, significantly more girls than boys.

Significant difference was observed between gender and achieved school success. Compared with girls, boys reported significantly moderate or low school success (Table 1).

**Table 1:** **Distribution of boys and girls and their differences according to demographic and socio-economic variables, Serbia**

**Variables**	**Total**	**Boys**	**Girls**	**Boys vs. Girls**
	**n (%)**	**n (%)**	**n (%)**	**p-value** ^*^
**Age**				.478
15–16	417 (61.1)	206 (62.4)	211 (59.8)	
17–19	266 (38.9)	124 (37.6)	142 (40.2)	
**Settlement**				.807
Non-urban	257 (37.6)	123 (37.3)	134 (38.0)	
Urban	426 (62.4)	207 (62.7)	219 (62.0)	
**Household wealth index**				.755
Poorest	83 (12.2)	44 (13.3)	39 (11.0)	
Poorer	140 (20.5)	65 (19.7)	75 (21.2)	
Middle class	129 (18.9)	59 (17.9)	70 (19.8)	
Richer	181 (26.5)	92 (27.9)	89 (25.2)	
Richest	150 (22.0)	70 (21.2)	80 (22.7)	
**Having one’s own room**				.615
No	189 (27.7)	88 (26.7)	101 (28.6)	
Yes	494 (72.3)	242 (73.3)	252 (71.4)	
**Family structure**				.603
Complete	604 (88.4)	294 (89.1)	310 (87.8)	
Incomplete^a^	79 (11.6)	36 (10.9)	43 (12.2)	
**School success**				<.001
Low/moderate	247 (36.2)	160 (48.5)	87 (24.6)	
High	436 (63.8)	170 (51.5)	266 (75.4)	

^a^Incomplete family structure: with one biological parent, be alone or with grandparents ^*^Chi-square test.

### Health risk behaviours

The prevalence of selected single health risk behaviour was computed separately for boys and girls. Boys more often than girls showed risky behaviour related to alcohol use, violence, sexual experience and smoking. On the contrary, girls more often reported taking tablets. Compared with girls, boys reported significantly currently using alcohol, bullying others, being sexual active during the last 12 months and having the first sexual experience before the age of 16 years (Table 2).

**Table 2:** **Distribution of boys and girls and their differences according to single health risk behaviours, Serbia**

**Variables**	**Boys**	**Girls**	**Boys vs. Girls**
	**n (%)**	**n (%)**	**p-value** ^*^
Alcohol use	95 (28.8)	49 (13.9)	.000
Bullying others	73 (22.1)	27 (7.6)	.000
Sexual activity	71 (21.5)	38 (10.8)	.000
Cigarette use	37 (11.2)	34 (9.6)	.531
Early sexual intercourse	31 (9.4)	13 (3.7)	.000
Tablets use	8 (2.4)	11 (3.1)	.370

^*^Chi-square test.

The percentage of adolescents (20.3%) was highest among those who reported one risk behaviour and decreased with increasing number of risk behaviours – it was lowest in the group of them who reported six risk behaviours. The distribution was similar in boys and girls. Boys more significantly reported two, three and four risk behaviours than girls (Table 3).

**Table 3:** **Distribution of boys and girls according to various models of health risk behaviours, Serbia**

**Variables**	**Total**	**Boys**	**Girls**	**Boys vs. Girls**
	**n (%)**	**n (%)**	**n (%)**	**p-value** ^*^
No risk	413 (60.4)	164 (49.7)	249 (70.5)	.000
One single risk	139 (20.3)	75 (22.7)	64 (18.1)	.003
Two risks	66 (9.6)	49 (14.8)	17 (4.8)	.000
Three risks	51 (7.5)	29 (8.8)	21 (5.9)	.009
Four risks	9 (1.3)	9 (2.7)	0 (0.0)	.000
Five risks	6 (0.9)	4 (1.3)	2 (0.6)	.181
Six risks	0 (0.0)	0 (0.0)	0 (0.0)	-

* Chi-square test or Fisher’s exact test.

Table 4 presents the distribution of boys and girls with two and more risk behaviours. Among boys, the most common two risk behaviours were alcohol use and bullying others while alcohol use and cigarette use were among girls. When considering the distribution of adolescents with three risk behaviours, boys were most often reported alcohol use, being sexually active and bullying others while, cigarette, alcohol use and sexual activity were the most common risk behaviours among girls. A small percentage of adolescents involved in more than three health risk behaviours.

**Table 4:** **Distribution of boys and girls with two and more health risk behaviours, Serbia**

**Variables**	**Total n (%)**	**Boys n (%)**	**Girls n (%)**
Alcohol use & bullying others	15 (2.2)	13 (4.0)	2 (0.6)
Alcohol use & sexual activity	12 (1.8)	11 (3.4)	1 (0.3)
Early sexual intercourse & sexual activity	10 (1.5)	6 (1.8)	4 (1.1)
Alcohol use & cigarette use	9 (1.3)	4 (1.2)	5 (1.4)
Sexual activity & bullying others	4 (0.6)	3 (0.9)	1 (0.3)
Tablets use & bullying others	3 (0.4)	3 (0.9)	0 (0.0)
Cigarette use & sexual activity	5 (0.7)	2 (0.6)	3 (0.8)
Cigarette use & bullying others	4 (0.6)	3 (0.9)	1 (0.3)
Alcohol use & tablets use	2 (0.3)	2 (0.6)	0 (0.0)
Alcohol use & cigarette use & sexual activity	12 (1.8)	5 (1.5)	7 (2.0)
Alcohol use & sexual activity & bullying others	10 (1.5)	8 (2.4)	2 (0.6)
Alcohol use & early sexual intercourse & sexual activity	9 (1.3)	6 (1.8)	3 (0.8)
Alcohol use & cigarette use & bullying others	6 (0.9)	4 (1.2)	2 (0.6)
Early sexual intercourse & sexual activity & bullying others	5 (0.7)	4 (1.2)	1 (0.3)
Alcohol use & tablets use & bullying others	2 (0.3)	0 (0.0)	2 (0.6)
Cigarette use & early sexual intercourse & sexual activity	3 (0.4)	1 (0.3)	2 (0.6)
Cigarette use & sexual activity & bullying others	1 (0.1)	1 (0.3)	0 (0.0)
Cigarette use & tablets use & bullying others	2 (0.3)	0 (0.0)	2 (0.6)
Cigarette & alcohol & early sexual intercourse & sexual activity	5 (0.7)	5 (1.5)	0 (0.0)
Cigarette & early sexual intercourse & sexual activity & bullying	2 (0.3)	2 (0.6)	0 (0.0)
Cigarette & Alcohol & early sexual intercourse & bullying others	1 (0.1)	1 (0.3)	0 (0.0)
Alcohol use & early sexual intercourse & sexual activity & bullying	1 (0.1)	1 (0.3)	0 (0.0)
Cigarette & alcohol & early sex & sexual activity & bullying others	5 (0.7)	4 (1.2)	1 (0.3)
Cigarette & alcohol & tablets & early sex & sexual activity	1 (0.1)	0 (0.0)	1 (0.3)
Cigarette & alcohol & tablets & early sex & sexual activity & bullying	0 (0.0)	0 (0.0)	0 (0.0)

### Sociodemographic factors and health risk behaviours

Table 5 presents the results of prevalence of concurrent multiple health risk behaviours among boys and girls by demographic characteristics and socio-economic status. The prevalence of multiple risk behaviours increased with age, living in urban environment, having one’s own room, living in incomplete family and achieving moderate or low school success, for both sexes.

**Table 5:** **Distribution of various models of health risk behaviours by demographic and socio-economic variables, Serbia**

**Variables**	**Boys**				**Girls**			
	**No risk**	**One risk**	**Two or more**	**p-value** ^*^	**No risk**	**One risk**	**Two or more**	**p-value** ^*^
	**n (%)**	**n (%)**	**n (%)**		**n (%)**	**n (%)**	**n (%)**	
**Age**				.000				.000
15–16	127 (61.6)	44 (21.4)	35 (17.0)		167 (80.2)	29 (13.7)	15 (7.1)	
17–19	37 (29.8)	31 (25.0)	56 (45.2)		82 (57.7)	35 (24.6)	25 (17.6)	
**Settlement**				.263				.453
Non-urban	68 (55.3)	23 (18.7)	32 (26.0)		96 (71.6)	27 (20.2)	11 (8.2)	
Urban	96 (46.4)	52 (25.1)	59 (28.5)		153 (69.9)	37 (16.9)	29 (13.2)	
**Household wealth index**				.086				.508
Poorest	24 (54.5)	12 (27.3)	8 (18.2)		29 (74.4)	7 (17.9)	3 (7.7)	
Poorer	40 (61.5)	9 (13.8)	16 (24.7)		57 (76.0)	13 (17.3)	5 (6.7)	
Middle class	28 (47.5)	13 (22.0)	18 (30.5)		49 (70.0)	11 (15.7)	10 (14.3)	
Richer	36 (39.1)	29 (31.5)	27 (29.4)		58 (65.2)	21 (23.6)	10 (11.2)	
Richest	36 (51.4)	12 (17.1)	22 (31.5)		56 (70.0)	12 (15.0)	12 (15.0)	
**Having one’s own room**				.299				.165
No	50 (56.8)	18 (20.5)	20 (22.7)		78 (77.2)	14 (13.9)	9 (8.9)	
Yes	114 (47.1)	57 (23.6)	71 (29.3)		171 (67.9)	50 (19.8)	31 (12.3)	
**Family structure**				.171				.001
Complete	145 (49.3)	71 (24.1)	78 (26.6)		226 (73.1)	55 (17.8)	28 (9.1)	
Incomplete^a^	19 (52.8)	4 (11.1)	13 (36.1)		23 (52.3)	9 (20.5)	12 (27.3)	
**School success**				.031				.014
Low/moderate	68 (42.5)	40 (25.0)	52 (32.5)		53 (60.9)	17 (19.5)	17 (19.5)	
High	96 (56.5)	35 (20.6)	39 (22.9)		196 (73.7)	47 (17.7)	23 (8.6)	

^a^Incomplete family structure: with one biological parent, be alone or with grandparents. ^*^Chi-square test.

The prevalence of multiple health risk behaviours was significantly higher in older boys and boys who achieved moderate or low school success than the younger ones (45.2% vs. 17.0%, p = .000), and those with high success in school (32.5% vs. 22.9%, p = .031). Among girls, there was a significant difference between age groups: older girls were more likely than the younger ones (17.6% vs. 7.1%, p = .000), family structure: girls from incomplete family were more likely than those from complete family (27.3% vs. 9.1%, p = .001), and school success: girls who achieved moderate or low school success were more likely than those with high success in school (19.5% vs. 8.6%, p = .014), to report multiple health risk behaviours (Table 5).

The results of multivariate logistic regression analyses of concurrent multiple health risk by demographic characteristics and socio-economic status are presented separately for boys and girls (Table 6). Multivariate logistic regression models showed a significant association of prevalence of concurrent multiple health risk behaviours with older age and with moderate or low school success in boys and girls and with incomplete family in girls and richer class households in boys. Adolescents who were older (OR = 5.82, boys; OR = 3.76, girls) and adolescents who achieved low or moderate (OR = 1.82, boys; OR = 3.36, girls) school success had significantly higher risk for concurrent multiple health risk behaviours than younger ones and those who achieved high school success. Also boys who came from a richer class households (OR = 3.14) and girls who live in incomplete family (OR = 5.07) had significantly higher risk for concurrent multiple health risk behaviours in comparison with boys from the poorest households and girls from complete family (Table 6).

**Table 6:** **Multivariate logistic regression of concurrent multiple health risk behaviours among boys and girls by demographic and socio-economic variables**

	**Two or more risk behaviours vs. no risk behaviours**			
**Variables**	**Boys n = (91 vs. 164)**		**Girls n = (40 vs. 249)**	
	**Adjusted OR**	**95% CI**	**Adjusted OR**	**95% Cl**
**Age**				
15–16	1		1	
17–19	**5.82** ^**^	**3.21–10.54**	**3.76** ^**^	**1.77–7.99**
**Settlement**				
Non-urban	1		1	
Urban	1.01	.51–1.99	1.32	.55–3.18
**Household wealth index**				
Poorest	1		1	
Poorer	1.31	.43–3.98	.76	.16–3.69
Middle class	2.22	.72–6.81	1.73	.39–7.63
Richer	**3.14** ^**^	**1.02–9.66**	1.45	.31–6.66
Richest	1.92	.60–2.67	1.93	.41–9.08
**Having one’s own room**				
No	1		1	
Yes	1.36	.69–2.67	1.60	.67–3.83
**Family structure**				
Complete	1		1	
Incomplete^a^	1.41	0.60–3.34	5.07**	**2.06–12.50**
**School success**				
Low/moderate	1		1	
High	1.82*	**1.02–3.26**	3.36**	**1.51–7.44**

^a^Incomplete family structure: with one biological parent, be alone or with grandparents. *Significantly different from reference group (P < 0.05). **Significantly different from reference group (P < 0.01).

Statistically significant associations are written in boldface.

## Discussion

Our study, however, showed that boys significantly more than girls demonstrated some single (currently using alcohol, currently smoking, being sexual active, having early sexual intercourse and bullying others) and concurrent multiple risk behaviours, which can be explained by the fact that such behaviour is often considered as socially acceptable for boys.

Previous studies on risk health behaviours by gender among adolescents have shown results similar to ours. The results of international cross sectional study “Health Behaviour in School-aged Children” (HBSC), showed clear evidence of differences by gender for risk-taking behaviour in almost all countries. Boys were more likely than girls to report they engage in risk behaviours on an experimental or regular basis. In the majority of countries, this was the case for alcohol and cannabis consumption, bullying and fighting. The patterns are less consistent for early sexual behaviour and smoking [8]. Results from the 2011 Youth Risk Behavior Survey (YRBS) indicated that among high school American students nationwide, the prevalence of having been in a physical fight, currently smoking, using alcohol and tablets were higher among male than female students, while older female students were more likely than male to report currently sexual activity [16]. The comparing of the results of the European School Survey Project on Alcohol and Other Drugs (ESPAD) on substance use among 15–16 year-old European students has shown that at the aggregate country level, with one exception — non-prescription use of tablets, slightly more boys than girls have reported having consumed alcohol and cigarettes in the past month, but the gender gap is very small between the 1995 and 2011 surveys [17]. Also, the results of two studies in China [6,18] have shown that boys were more likely than girls to report they engage in multiple health risk behaviours.

In general, many previous studies have confirmed the clustering of harmful lifestyles among adolescents [19-22]. Our study showed that the prevalence of multiple health risk behaviours significantly increased with age for both sexes. Similar results have been found in study conducted in Japan and the United States [23,24], while some other studies have shown that the prevalence of multiple risks increased with age for boys, but not for girls [6,25].

In our study, boys and girls who were older and those who achieved low or moderate school success, boys from richer-class households and girls from incomplete family had significantly higher risk than those with high school success, boys from the poorest households and girls from complete family. The results of previous studies showed that living in an incomplete family [26-28], parental education [29,30] and family affluence [8,25,31] were associated with alcohol consumption. Weekly drinking tended to be more commonly reported among boys from high family affluence in some countries but in only a few for girls [8].

Also, the national study has shown an association between incomplete families [30] and smoking habits. On the contrary, results of the studies was conducted in a wide range of European countries, the US, Canada and Israel have showed that family affluence was not statistically associated with regular smoking in most countries [8,32].

The results from the 2009/2010 HBSC survey indicated that adolescents from less affluent families were more likely to smoke weekly in a minority of countries. Prevalence of experience of sexual intercourse was significantly lower among boys in high-affluence families in around a quarter and higher in only three, while for girls it increased with higher affluence in a few. Bullying others was linked to higher affluence in eastern countries and lower affluence in other regions [8].

This can be explained by the fact that adolescents from affluent families grow up in families that have only outward form of family (large apartment in which they live together) but not the classical form of internal closeness, connectedness and familiarity. Their parents are committed to their careers, acquiring material wealth and staying a little time at home. They are under increasing pressure to achieve excellent performance in school, in many extracurricular activities and social life. This way of life has a negative impact on adolescents’ health. Where family affluence was not a significant influence on risk behaviour, it may be that other social influences arising from the family, peers and school had a greater impact during adolescence [33].

This analysis has several limitations. First, the cross-sectional design makes it difficult to determine the direction of causality and the fact that the resulting link does not necessarily reflect the relationship between sociodemographic factors and any risky behaviour and risky behaviour among themselves. This limitation can be overcome with the use of longitudinal studies. A second methodological issue is with regard to the accuracy of the data collected through self-administered questionnaire. Consequently, the bias in the provision of data, is influenced on the one hand of sex of respondents (young men often exaggerate their experience while the girls reduced or diminished their experience) and on the other side of the sensitivity of the person due to past experiences related with certain risk behaviours. Finally, it should be mentioned that this study is based on eight year old survey and relates to a specific period of time and social context in Serbia. Despite the ongoing socio - economic recovery of the country, the citizens of Serbia, during this period, still felt the consequences of the nineties that were marked by wars, sanctions and the collapse of all segments of society, including health care.

However, this is the first study investigating the prevalence of concurrent health risk behaviours and its association with demographic and socio-economic determinants of health among adolescents in Serbia and this fact creates a possibility to repeat this research with the same methodology and study design in order to follow up the trend of risk behaviours indicators over time. It could be used to estimate the effects of work on the reform and development of health systems Serbia and preserving and improving the health of adolescents, as well as in the creation of health policies whose implementation would contribute to reducing health inequalities.

## Conclusions

This study has shown association between demographic characteristics and socio-economic status with multiple health risk behaviours among adolescents in Serbia. These findings should be an integral part of further preventive interventions which should be gender and age specific, oriented towards older adolescents and adolescents who have moderate or low school success, boys from richer class households and girls who live in incomplete families.

## Abbreviations

GDP: The gross domestic product
Wealth Index: The demographic and health survey wealth index
SD: Standard deviation
OR: Odds ratio
Cl: Confidence Interval
HBSC: Health behaviour in school-aged children
YRBS: Youth risk behavior survey
ESPAD: The European school survey project on alcohol and other drugs
FAS: The family affluence scale
